# Case Report: *MDFIC* gene mutation resulting in central conducting lymphatic anomaly facilitates group A *Streptococcus* sepsis

**DOI:** 10.3389/fped.2024.1367532

**Published:** 2024-09-25

**Authors:** Johannes Weidner, Kai Fiedler, Mechthild Schulze-Becking, Christiaan Peter Sentner, Christoph Korenke, Axel Heep

**Affiliations:** ^1^ Department of Pediatric Surgery, Hannover Medical School, Hannover, Germany; ^2^ School VI—School of Medicine and Health Sciences, Carl von Ossietzky Universität Oldenburg, Oldenburg, Germany; ^3^ Section of Neonatology and Pediatric Intensive Care, Department of Pediatrics, School VI—School of Medicine and Health Sciences, Carl von Ossietzky Universität Oldenburg, Oldenburg, Germany; ^4^ Section of Pediatric Neurology, Department of Pediatrics, School VI—School of Medicine and Health Sciences, Carl von Ossietzky Universität Oldenburg, Oldenburg, Germany

**Keywords:** case report, *MDFIC* mutation, central conducting lymphatic anomaly, sepsis, group A *Streptococcus* infection

## Abstract

**Background:**

Central conducting lymphatic anomaly (CCLA) is a heterogeneous disorder characterized by structural anomalies in the main collecting lymphatic vasculature. These anomalies result in chronic chylous leaks, causing issues such as congenital hydrothorax and potentially impairing the normal immune response. Recently, mutations in the MyoD family inhibitor domain-containing (*MDFIC*) gene have been identified as a cause of CCLA. Group A *Streptococcus* infections are common, and timely identification of patients at risk for severe complications is crucial.

**Case presentation:**

Here, we present the case of a 13-year-old female patient with CCLA associated with an *MDFIC* mutation, who suffered from a severe group A *Streptococcus* sepsis. Initially, the patient was unresponsive to aggressive fluid resuscitation. Although the course of the sepsis was severe, standardized treatment according to the surviving sepsis campaign proved effective in stabilizing the patient.

**Discussion:**

The patient's *MDFIC* mutation may have contributed to the severe clinical course of the sepsis. It is theorized that this mutation affects the function of the immune system both indirectly, by causing CCLA, and directly, by potentially influencing transcriptional activity in immune cells. More research on the effect of *MDFIC* mutations on immune responses is required.

## Introduction

Central conducting lymphatic anomaly (CCLA) is a heterogeneous group of congenital malformations of the main collecting lymphatic vessels, resulting in chylothorax, chylous ascites and lymphedema. Here, we present the case of a patient with CCLA based on a rare homozygous mutation of the MyoD Family Inhibitor Domain-Containing protein (*MDFIC*) gene (1). Group A *Streptococcus* (GAS) infections are common and manifest a broad spectrum of diseases, from asymptomatic colonization to severe septicemia. Most GAS infections can be effectively treated with β-lactam antibiotics (2). In 2022, there was an increased incidence of invasive GAS (iGAS) infections (3). In the case presented, the *MDFIC* mutation and the resulting CCLA possibly facilitated the development of severe iGAS sepsis by impairing the immune response of the patient.

## Case report

We present the case of a 13-year-old girl who was initially admitted to the hospital with severe sepsis and required transfer to a larger facility due to her worsening condition. One week prior to the admission, the patient presented to her general practitioner with an upper airway infection, but she did not adhere to the prescribed antibiotic therapy.

The patient was born at 37 + 1 weeks by caesarian section, which was carried out due to progressing bilateral pleural effusions identified in the 35th gestational week. The chylous effusions were treated by postnatal pleural puncture. In addition, she was diagnosed with a muscular ventricular septal defect. In the following years, she was seen regularly in our outpatient clinic for recurrent lymphedema in her feet and lower extremities, as well as recurring pleural effusions. At the age of 11, she was diagnosed with a homozygous stop mutation in *MDFIC* gene. Mutations in this gene are known to be associated with CCLA. Despite the mutation and the resulting CCLA, she reached all development milestones as expected. Prior to the development of GAS sepsis described in this case report, our patient did not exhibit signs of increased susceptibility to infections.

During the acute admission for sepsis, the patient presented in a severely reduced physical state. Her Mean arterial pressure (MAP) was reduced at 48 mmHg, with a prolonged capillary refill time of 3–4 s. The lungs were clear to auscultation, with slightly reduced breathing sounds over the left lung and edema in both feet. Laboratory tests showed leucopenia (3.31 × 10^9^/L), a C-reactive protein (CRP) level of 151.0 mg/dl, and a Procalcitonin (PCT) level of 82.1 µg/L. Serum immunoglobin levels were within normal range. Blood gas analysis revealed acidosis with a pH of 7.33 and lactate of 4.9 mmol/L. Ultrasound examination showed free fluid in the abdomen, and a chest x-ray revealed bilateral pleural effusions. Echocardiography revealed a small pericardial effusion (5 mm).

**Figure 1:**
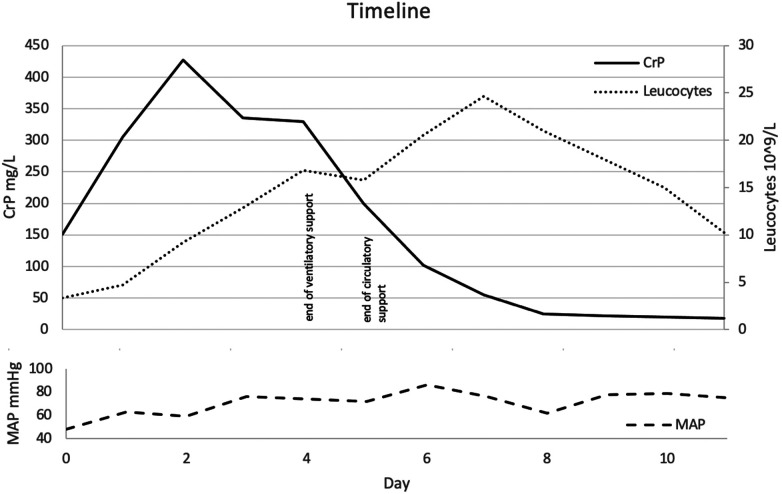
Timeline presented as day after admission. Course of pro-inflammatory markers and noticeable clinical hallmarks.

The patient was admitted to the intensive care unit, where she received intravenous fluid therapy that temporarily stabilized her blood pressure. Catheters were inserted to gain central venous access and allow continuous arterial blood pressure monitoring. Vasopressor therapy with norepinephrine and dobutamine was administered. After taking blood cultures, a calculated antimicrobial regimen with penicillin G, ceftriaxone, and clindamycin was started. Blood cultures indicated a *Streptococcus pyogenes* infection, sensitive to the administered antibiotics. Unexpectedly, the leucocyte count only exceeded the upper limit of normal on the third day of admission. For a detailed course of the infectious parameters, see Figure 1.

On day 1 after admission, the patient developed severe tachypnea, requiring high-flow nasal cannula oxygen therapy with intermittent continuous positive airway pressure therapy. In addition, radiological findings suggested lung edema, which was treated with diuretics. Over the following days, both clinical and radiological findings improved to the point that the ventilatory support could be weaned and eventually stopped (Figure 1).

The patient was discharged after 11 days. We recommended lymphatic drainage, physiotherapy, and further immunological assessment.

## Discussion

CCLA is described as an insufficiency of the thoracic duct, which leads to lymphatic reflux and subsequent accumulation of chylous fluids (4). *MDFIC* mutations have been reported to influence the development of diseases of the lymphatic vasculature. Patients typically present with signs such as recurring lymphedema and chylous serosal effusions (1).

*MDFIC* mRNA expression has been detected in many tissues, including leucocytes, lymph nodes, and the spleen (5). A recently published mouse model (homozygous *Mdfic* mutant mice) showed that mutations in this gene affect the formation of lymphatic vessels and their valves, resulting in a phenotype resembling that of patients with CCLA. Given the central role of the lymphatic system in regulating tissue fluid homeostasis, dietary lipid absorption, and immune cell trafficking, patients with *MDFIC* mutations might be more susceptible to fulminant iGAS and sepsis. Deficient immune cell trafficking could explain our observation of the delayed increase of leucocyte concentrations in peripheral blood samples (Figure 1). While defective lymph flow has been linked to decreased immune function and increased susceptibility to infections, data on exact pathomechanisms remain pending (6).

Peripheral blood mononuclear cells of a patient with a frameshift mutation of *MDFIC* have been found to display a negative interferon signature to Lipopolysaccharid (LPS) treatment. The authors concluded that interferon signature-mediated inflammatory responses might be negatively affected by this mutation. These findings suggest an additional influence of the *MDFIC* mutation on cellular immune responses (7). Other data generated from whole blood samples of patients with septic acute kidney injury (SAKI) identified *MDFIC* as one of the 10 hub genes involved in the regulatory network of SAKI (8).

To our knowledge, there is only one other reported case of a child with an *MDFIC* mutation who died after a *S. pyogenes* infection. A causal connection cannot be inferred based on two cases. More research is warranted on this topic (1).

Therapeutic options for CCLA are scarce. Although sirolimus, an inhibitor of the mammalian target of rapamycin (mTOR) acting as a kinase regulating cell metabolism and growth, has been shown to positively affect other vascular anomalies, this does not hold true for CCLA. Data on volumetric measurements did not show a significant reduction in size. In general, CCLA treatment is considered complicated and mostly surgical (9–11).

Surgical correction of lymphatic anomalies by lymphaticovenous bypassing has proven successful in 50% of patients with severe manifestations of CCLA. After assessing technical success in all cases, Taghinia et al. concluded that additional functional factors might influence the treatment success. Treatment failure could be explained by dysfunctional lymphatic valves based on an *MDFIC* gene mutation. Whether patients benefit from lymphaticovenous bypassing in the long term remains unknown (12).

Throughout the follow-up period, the patient remained in good health, attending regular follow-up appointments at our clinic. Results from immunological investigations are still pending. Intranodal dynamic magnetic resonance lymphangiography, along with the clinical presentation, is planned in the future to evaluate the eligibility for surgical correction of the lymphatic anomaly (4).

## Conclusion

In conclusion, we can say that patients with *MDFIC* mutations seem more susceptible to fulminant courses of infectious diseases, and vigorous treatment should be considered upon clinical signs of invasive infections. Sepsis therapy, according to surviving sepsis guidelines, proved effective in treating our patient. Further research on the effect of lymphatic malformations on immune responses, including subtype analysis of differing *MDFIC* mutations, is warranted to improve the management and outcomes of potentially life-threatening infections in these patients.

## Data Availability

The original contributions presented in the study are included in the article/Supplementary Material, further inquiries can be directed to the corresponding author.
